# Transcriptional Targeting of Mature Dendritic Cells with Adenoviral Vectors via a Modular Promoter System for Antigen Expression and Functional Manipulation

**DOI:** 10.1155/2016/6078473

**Published:** 2016-06-29

**Authors:** Ilka Knippertz, Andrea Deinzer, Jan Dörrie, Niels Schaft, Dirk M. Nettelbeck, Alexander Steinkasserer

**Affiliations:** ^1^Department of Immune Modulation at the Department of Dermatology, Universitätsklinikum Erlangen, Hartmannstrasse 14, 91052 Erlangen, Germany; ^2^Department of Dermatology, Universitätsklinikum Erlangen, Hartmannstrasse 14, 91052 Erlangen, Germany; ^3^German Cancer Research Center, Im Neuenheimer Feld 280, 69120 Heidelberg, Germany

## Abstract

To specifically target dendritic cells (DCs) to simultaneously express different therapeutic transgenes for inducing immune responses against tumors, we used a combined promoter system of adenoviral vectors. We selected a 216 bp short Hsp70B′ core promoter induced by a mutated, constitutively active heat shock factor (mHSF) 1 to drive strong gene expression of therapeutic transgenes MelanA, BclxL, and IL-12p70 in HeLa cells, as well as in mature DCs (mDCs). As this involves overexpressing mHSF1, we first evaluated the resulting effects on DCs regarding upregulation of heat shock proteins and maturation markers, toxicity, cytokine profile, and capacity to induce antigen-specific CD8^+^ T cells. Second, we generated the two-vector-based “modular promoter” system, where one vector contains the mHSF1 under the control of the human CD83 promoter, which is specifically active only in DCs and after maturation. mHSF1, in turn, activates the Hsp70B′ core promotor-driven expression of transgenes MelanA and IL-12p70 in the DC-like cell line XS52 and in human mature and hence immunogenic DCs, but not in tolerogenic immature DCs. These* in vitro* experiments provide the basis for an* in vivo* targeting of mature DCs for the expression of multiple transgenes. Therefore, this modular promoter system represents a promising tool for future DC-based immunotherapies* in vivo*.

## 1. Introduction

Dendritic cells (DCs) function as sentinels at the interface of the innate and the adaptive immune system, thereby inducing highly potent and antigen-specific immune responses triggered by “danger” signals. As master antigen-presenting cells (APCs), they not only express proinflammatory cytokines (particularly IL-12) but also capture, process, and present antigens on major histocompatibility (MHC) molecules to naïve T cells, leading to an adaptive T cell-mediated immune response [1]. Importantly, DCs possess the unique feature to cross-present typical MHC class II restricted antigens via MHC class I, thereby inducing cytotoxic CD8^+^ cell (CTL) responses and enhancing antitumor humoral responses [2, 3]. In this context, DCs have been shown to have a significant impact on oncogenesis, tumor progression, and response to therapy in various preclinical tumor models and preclinical studies [4]. Additionally, the discovery of immune checkpoint blockers (e.g., monoclonal antibody [mAb] to CTLA-4; mAb to PD-1) and their possible combination with DC vaccination has made cancer immunotherapy one of the most exciting topics in the oncology field recently [5, 6]. The main DC-based anticancer interventions developed so far comprise various* ex vivo* and* in vivo* immune manipulating strategies*. Ex vivo*, DCs were loaded with different tumor associated antigens (TAAs) using various strategies including (i) peptide or protein pulsing, (ii) loading with complete tumor lysate or (iii) tumor apoptotic bodies, and (iv) RNA transfection or (v) viral transduction [7].* In vivo*, cancer antigens can be delivered to DCs by fusing them to monoclonal antibodies specifically targeting DC surface receptors such as mannose receptor, C type 1 (MRC1), CD209 (DC-SIGN), or DEC-205 [7–10]. Another strategy is to incorporate TAAs, DNA, RNA, or toll-like receptor ligands into DC-targeting immunoliposomes, nanoparticles, or viral vectors for delivery [11–14].

In recent years, viral vectors like lentiviruses or adenoviruses (Ads) have been widely used in clinical trials for many different types of inherited or acquired disorders with Ad vectors being the ones most commonly used for cancer gene therapy [15, 16]. Adenoviruses have many advantages as they (i) can be grown into high titer stable stocks, (ii) infect nondividing and dividing cells of different types, (iii) are maintained in cells as an episome, and (iv) have been proven safe and well tolerated while also being therapeutically active [16]. Most Ad vectors are genetically modified versions of Ad5 and can be either replication-defective or replication-competent. Replication-defective Ad vectors are effectively used as gene delivery vehicles and have the essential E1A, E1B, and E3 genes deleted and replaced by an expression cassette encoding for foreign therapeutic transgenes up to ~6.5 kb in size [16]. Specific targeting of a cell (e.g., a DC) can be achieved by transductional or transcriptional targeting or a combination of both. Transductional targeting involves chemically or genetically modifying an adenovirus to redirect its tropism from its natural binding partner CAR (coxsackie and adenovirus receptor) to a new target expressed preferentially on the target cell like, for example, DC-SIGN or DEC-205 for DCs [17]. Transcriptional targeting, on the other hand, involves using tissue-specific promoters to genetically limit expression of the introduced gene to distinct tissues [17].

However, there was no cell type- and maturation-specific promoter available for DCs until recently. In 2013, we identified and characterized a tripartite promoter complex specifically regulating human CD83 expression in mature immunogenic DCs. We found that it consisted of a 261 bp core promoter flanked by (i) a 164 bp upstream regulatory element and (ii) a 185 bp downstream enhancer separated from the promoter by a 500 bp noncoding spacer sequence [18]. All three elements were shown to be essential for transcriptional activation in mature immunogenic DCs, while not mediating this specific activation in tolerogenic immature DCs (iDCs), or other CD83-positive cell types, such as subsets of activated B or T cells. Thus, this promoter complex shows great potential for the transcriptional targeting of mature DCs and thereby the development of new immunotherapeutic approaches.

Despite the successes of DC-based immunotherapy in individual patients, the immunogenic potential to induce effective antitumor CTL responses is still considered suboptimal [19]. Furthermore, the* ex vivo* generation of DC-vaccines is laborious and expensive. Hence, new vaccination strategies involving* in vivo* targeting of DCs for antigen expression and functional manipulation should be addressed. To do this, we developed a combined promoter system to transcriptionally target human DCs to express several therapeutic transgenes at the same time, the “modular promoter (MP)” system. Due to the limited space for foreign DNA in adenoviral vectors, it is problematic to use large, cell-specific promoters for several transgenes. Therefore, we combined the cell type- and maturation-specific CD83 promoter, which has a size of 1.2 kb [18], with another short and induction-specific promoter in a two-vector system. In this system, the transgenes in one vector are under the control of a short inducible promoter, which is activated by a factor, expressed from the larger, highly specific CD83 promotor in the second vector. As a short, inducible promoter we chose the short heat shock protein (Hsp) 70B′ promoter, which has been reported before to mediate specifically heat-dependent transgene expression in replication-deficient adenoviruses [20]. The* hsp70B*′ gene, along with* hsp70(A)-1*,* hsp70(A)-2,* and* hsp70B, *belongs to the* hsp70* gene family, all regulated by the heat shock transcription factor 1 (HSF1) [20–23]. HSF1 is a highly conserved transcription factor that coordinates stress-induced transcription and directs versatile physiological processes in eukaryotes [24]. Upon induction, it undergoes trimerization, as well as phosphorylation, followed by nuclear translocation and DNA binding to heat shock promoters [25].

For our MP system we used a mutated, constitutively active HSF1 (mHSF1) [26] whose expression is controlled here by the DC- and maturation-specific human CD83 promoter [18]. In turn, mHSF1 then binds to the short heat shock response element Hsp70B′ driving the simultaneous expression of multiple therapeutic transgenes. Concomitantly, mHSF1 also binds to endogenous heat shock promoters of targeted DCs. We have shown previously that exposure of human DCs to thermal stress leads to an upregulation of Hsp70A, costimulatory molecules, and proinflammatory cytokines, as well as a markedly improved capacity to prime autologous naïve CD8^+^ T cells* in vitro* [27]. Therefore, in the present study we also analyzed the effects of mHSF1 overexpression on DCs.

Our results demonstrate that the newly generated MP system allows, for the first time, specific and simultaneous expression of different therapeutic transgenes in human mature DCs* in vitro*, representing a promising tool to improve future DC-based immunotherapies. Moreover, we found no effects regarding the viability, maturation, and function of DCs by overexpressing mHSF1.

## 2. Methods

### 2.1. Human Dendritic Cells and CD8^+^ T Cells

Generation of human monocyte-derived dendritic cells (DCs) was performed as previously described [28]. In brief, peripheral blood mononuclear cells (PBMCs) were prepared from leukoreduction system chambers (LRSCs) of healthy donors by density centrifugation, followed by plastic adherence on tissue culture dishes (BD Falcon, MA, US). The nonadherent fraction was stored at −80°C for isolation of autologous CD8^+^ T cells, while the adherent cell fraction was cultured for 4 days in DC-medium consisting of RPMI 1640 (Lonza, Basel, Switzerland) supplemented with 1% (vol/vol) of heat-inactivated human serum type AB (Lonza), 1% Penicillin/Streptomycin/L-Glutamine (Sigma-Aldrich, St. Louis, MO, US), and 10 mM Hepes (Lonza) as well as 800 IU/mL (day 0) or 400 IU/mL (day 3) recombinant human granulocyte macrophage colony-stimulating factor (GM-CSF) and 250 IU/mL (day 0 and 3) recombinant IL-4 (both Miltenyi, Bergisch-Gladbach, Germany). On day 4, immature DCs (iDCs) were used for further experiments. Maturation of DCs was induced by the addition of a maturation cocktail (MC) consisting of 200 U/mL IL-1*β*, 1000 U/mL IL-6 (both CellGenix, Freiburg, Germany), 10 ng/mL TNF-*α* (Beromun; Boehringer Ingelheim, Germany), and 1 *μ*g/mL PGE_2_ (Prostin E_2_, Pfizer, NY, US) for 24 or 48 hours or by 0.1 ng/mL lipopolysaccharide (LPS; Sigma-Aldrich) for 20 hours. For the isolation of autologous CD8^+^ T cells the nonadherent fraction was thawed and CD8^+^ T cells were isolated using anti-CD8 MACS beads (Miltenyi) according to the manufacturer's instructions. Afterwards cells were cultured in RPMI 1640 (Lonza) medium additionally containing 10% (vol/vol) of heat-inactivated human serum type AB (Lonza), 1% L-Glutamine (Sigma-Aldrich), 20 mg/L gentamycin, 10 mM Hepes (Lonza), 1 mM sodium pyruvate (Lonza), and 1% MEM nonessential aa (PAN Biotech, Aidenbach, Germany). Cryopreservation and thawing of CD8^+^ T cells were performed as described elsewhere [29]. Cell counting was performed by using a Neubauer counting chamber and Trypan Blue for exclusion of dead cells. Whenever relevant, HLA-A0201^+^ donor material was used.

### 2.2. Approvals and Legal Requirements

For the generation of PBMCs, moDCs, and CD8^+^ T cells from LRSCs of healthy donors, the positive vote from the local ethics committee was obtained (ethics vote number 4556).

### 2.3. Cell Lines

HeLa cells were cultured in Dulbecco's Modified Eagle Medium (DMEM; Lonza) supplemented with 10% (vol/vol) FCS (PAA/GE Healthcare, Little Chalfont, UK) and 1% (vol/vol) Penicillin/Streptomycin/L-Glutamine (Sigma-Aldrich). XS52 cells, kindly provided by A. Takashima (University of Texas Southwestern Medical Center, Dallas, TX, US), were cultured in Iscove's Modified Dulbecco's Medium (IMDM; Lonza) supplemented with 10% (vol/vol) FCS, 1% (vol/vol) Penicillin/Streptomycin/L-Glutamine, 1% (vol/vol) sodium pyruvate (Lonza), 10% (vol/vol) NS47 supernatant, and 10 ng/mL murine GM-CSF. 293 (Quantum) cells were cultured in RPMI 1640 (Lonza) supplemented with 10% (vol/vol) FCS and 1% (vol/vol) Penicillin/Streptomycin/L-Glutamine.

### 2.4. Plasmid Vectors

The promoterless pGL3-Basic luciferase reporter vector (Promega, WI, US) was used for determination of vector-related background activity and to generate pHsp70B′_−29/−489_ and pHsp70B′_−29/−242_ by digesting the human* hsp70B* gene 5′-region (according to GenBank accession no. X13229) with HindIII/BamHI or HindIII/SmaI, respectively. pHsp70B′_−29/−242_ was then used to generate pMelA, pBclxL, and pIL-12 by replacing the luciferase gene by the open reading frame sequences of either MelanA/MART-1, Bcl-xL, or the human single-chain of IL-12(p70) [30] (kindly provided by F. Schnieders, Provecs Medical GmbH, Hamburg, Germany). The vector pMelA/BclxL/IL-12 was then generated by the sequential connection of the expression cassettes Hsp70B′_−29/−242_-MelanA/, Hsp70B′_−29/−242_-BclxL/, and Hsp70B′_−29/−242_-IL-12(p70). Plasmids expressing mHSF1 under the control of the human CD83 promoter (P-510) were manufactured by replacing the luciferase gene by the open reading frame sequence of mHSF1 [26] (kindly provided by R. Voellmy, HSF Pharmaceuticals, Fribourg, Switzerland) of pGL3-CD83 promoter constructs described before [18], resulting in pP-510/mHSF1, pEs/P-510/mHSF1, and pEas/P-510/mHSF1. All constructs were generated by standard cloning procedures.

The pGL3-Promoter vector (Promega), containing a SV40 promoter, was used as a positive control and to determine transfection efficacy.

All plasmids for transient transfection experiments were purified by standard endo-free anion-exchange columns (Qiagen, Hilden, Germany) and verified by DNA sequencing (MWG Biotech, Ebersberg, Germany).

### 2.5. Recombinant Adenoviruses

Ad5MelA/BclxL/IL-12, Ad5MP2, Ad5mHSF1, Ad5P-510/mHSF1, Ad5Es/P-510/mHSF1, Ad5Eas/P-510/mHSF1, Ad5MelA, Ad5Luc1, and Ad5TL are first generation, E1- and E3-deleted, replication-deficient adenoviral vectors. Ad5mHSF1 contains mHSF1 [26] under the control of a CMV promoter, kindly provided by R. Voellmy (HSF Pharmaceuticals, Fribourg, Switzerland). Ad5Luc1 contains a CMV-firefly luciferase cassette and Ad5TL contains both a CMV-firefly luciferase cassette and a CMV-GFP cassette (both kindly provided by D. T. Curiel, Washington University School of Medicine, MO, US). All other replication-deficient adenoviruses were cloned as follows: a gene cassette containing either a Hsp70B′_−29/−242_-MelanA/Hsp70B′_−29/−242_-BclxL/Hsp70B′_−29/−242_-IL-12(p70)-, a Hsp70B′_−29/−242_-MelanA/Hsp70B′_−29/−242_-IL-12(p70)- (MP2), a P-510-mHSF1-, Es/P-510-mHSF1-, Eas/P-510-mHSF1-, or a CMV-MelanA sequence was inserted into pShuttle. Virus genomes were obtained by homologous recombination of the corresponding shuttle plasmids containing the different expression cassettes indicated above with pAdEasy-1 in* E. coli* BJ5183 as described before [31]. Adenovirus particles were produced by transfection of the different PacI-digested pAd vectors into 293 cells using Lipofectamine (Invitrogen/Life Technologies, CA, US). All viruses were amplified in 293 cells and purified by two rounds of CsCl equilibrium density gradient ultracentrifugation. Verification of viral genomes and exclusion of wild-type contamination were performed by PCR. Physical particle concentration [viral particles (vp)/mL] was determined by OD_260_ reading and infectious particle concentration was determined by TCID_50_ assay on 293 cells.

### 2.6. Transfection of Cell Lines

For transfection of HeLa cells, 5 × 10^4^ or 8 × 10^4^ cells were seeded in a 24-well or 12-well plate (BD Falcon) in a final volume of 500 *μ*L or 1 mL growth medium per well, respectively. The next day, HeLa cells were transfected using* Lipofectamine™* LTX with* Plus™* reagent (Invitrogen/Life Technologies) according to the manufacturer's protocol with a total amount of 0.5 *μ*g (24-well) or 0.7 *μ*g (12-well) DNA. Twenty-four hours later, cells were transduced with adenoviruses at 300 TCID_50_/cell in a final volume of 200 *μ*L DMEM (Lonza) containing 2% FCS (PAA/GE Healthcare). After 1.5 hours of incubation at room temperature on a rocker, 800 *μ*L (24-well) or 2 mL (12-well) growth medium was added. Cells were harvested for further experiments one day after transduction.

For transfection of XS52 cells, 5 × 10^5^ cells were seeded in a 6-well plate (BD Falcon) in a final volume of 2 mL cell growth medium per well. The following day, cells were transduced using X-tremeGENE*™* HP DNA Transfection Reagent (Roche, Basel, Switzerland) according to the manufacturer's instructions with 2 *μ*g DNA and 6 *μ*L transfection reagent in a final volume of 200 *μ*L Opti-MEM I Reduced Serum Medium (Gibco/ThermoFisher Scientific, MA, US). Four hours after transfection, 2 mL of growth medium was added and cells were incubated for another 24 hours at 37°C/5% CO_2_ before they were used for further analyses.

### 2.7. Adenoviral Transduction of Dendritic Cells

Immature d4 DCs were seeded in 12-well tissue culture plates (BD Falcon) at a concentration of 1 × 10^6^ cells/well in 250 *μ*L medium supplemented with either 800 U/mL GM-CSF and 500 U/mL IL-4 (for iDCs) or the maturation cocktail consisting of 400 U/mL IL-1*β*, 2000 U/mL IL-6, 20 ng/mL TNF-*α*, and 2 *μ*g/mL PGE_2_, in addition to IL-4 and GM-CSF (for mDCs). When indicated, 0.1 ng/mL LPS were used instead of the cytokine cocktail, which was added to the cell culture after the transduction. Adenovirus was added to the cells at 500 TCID_50_/cell in a final volume of 250 *μ*L medium without cytokines. For cotransduction of two adenoviruses, the final volume was halved to 125 *μ*L medium without cytokines, also resulting in a final infection volume of 500 *μ*L. After 1.5 hours of incubation at room temperature, 2 mL of growth medium replenished with cytokines as described before was added per well. To determine transduction efficacy, cells were transduced with Ad5TL and the percentage of living green fluorescent cells was assessed by flow cytometric analysis with a FACScan cell analyzer (BD Biosciences, NJ, US). Only experiments that yielded transduction efficiencies of more than 70% were evaluated and are shown.

### 2.8. Transfection and Peptide Pulsing of Dendritic Cells

Immature DCs were transduced with Ad5Luc1 or Ad5mHSF1 or medium-treated in the presence of the cytokine maturation cocktail as described above. The next day, transfection of mature DCs with MelanA/MART-1 RNA was performed using the nonlipid cationic reagent Transmessenger*™* transfection kit (Qiagen) following an adapted protocol from Liao et al. [32] as described previously in detail by Schaft et al. [33]. For peptide pulsing, mature DCs were incubated with 10 *μ*g/mL of the MelanA-derived HLA-A2-binding analogue peptide ELAGIGILTV in DC cell culture medium for 1 hour at 37°C/5% CO_2_.

### 2.9. Priming and Tetramer Staining of CD8^+^ T Cells

MelanA RNA-transfected or MelanA peptide-loaded mature DCs were used to stimulate autologous MACS-sorted CD8^+^ T cells at a ratio of 1 : 10 for one week at 37°C/5% CO_2_. Fresh T cell medium was added when necessary and on days 2 and 4 1000 IU/mL IL-2 (Novartis, Nürnberg, Germany) and 10 ng/mL IL-7 were supplemented. On day 7, cells were harvested and stained with a HLA-A2-MelanA/iTag MHC class I-tetramer (Beckmann Coulter, Krefeld, Germany) in combination with an anti-CD8-PC7 antibody (Beckmann Coulter). Finally, cells were analyzed using a FC500 cytofluorometer (Beckmann Coulter).

### 2.10. Flow Cytometric Analyses

Cells were stained with specific mAb or appropriate isotype controls for 30 min at 4°C in FACS buffer (Dulbecco's PBS [Lonza] containing 2% FCS [PAA/GE Healthcare]), washed twice, and finally resuspended in cold FACS buffer containing 0.1 *μ*g/mL propidium iodide (PI) (Carl Roth, Karlsruhe, Germany). Stained cells were immediately analyzed with a FACScan cell analyzer (BD Biosciences). Cell debris and dead cells were excluded from the analyses by gating on proper forward and sideward light scatter and on PI negative cells. Also, percentages of PI positive cells were calculated using this gating strategy. A minimum of 10^4^ living cells was measured for each sample and results were analyzed using FCS Express 4 Flow Cytometry Software (De Novo Software, CA, US). The following monoclonal antibodies (all from BD Biosciences) were used to determine the phenotype of DCs: PE-labeled mouse anti-human CD25 (M-A251), CD80 (L307.4), CD83 (HB15e), CD86 (IT2.2), HLA-ABC (G46-2.6), and HLA-DR (G46.6). Isotype mAb controls (all from BD Biosciences) used but not shown were IgG1-PE (MOPC-21), IgG2a-PE (G155-178), and IgG2b-PE (27-35).

### 2.11. Cytometric Bead Array

Inflammatory cytokines secreted by DCs into the supernatant were assessed using the Human Inflammatory Cytokine Cytometric Bead Array (CBA; BD Biosciences) according to the manufacturer's protocol.

### 2.12. ELISA

IL-12p40 and IL-12p70 concentrations in cell culture supernatants were determined by standard two-site sandwich BD OptEIA*™* Human IL-12 (p70) and Human IL-12 (p40) ELISA Kits (BD Biosciences) according to the manufacturer's manual.

### 2.13. BCA Protein Assay

Protein concentrations of cell lysates for SDS-PAGE and luciferase reporter assays were determined by Pierce*™* Protein Assay Kit (ThermoFisher Scientific, MA, US) according to the manufacturer's protocol.

### 2.14. SDS-PAGE and Western Blotting

Whole cell extracts for SDS-PAGE were generated as follows: 1 × 10^6^ DCs or XS52 cells were washed with ice-cold PBS and then resuspended in 50 *μ*L lysis buffer mixed with NaVO_3_, NaF, and PMSF. Afterwards, protein concentrations were determined by the BCA protein assay described above. To prepare sample proteins, they were boiled in the presence of a loading dye mixed with SDS and 2-mercaptoethanol for 10 min at 95°C. Then, 20 to 30 *μ*g of total protein per sample was separated on a 12.5% polyacrylamide gel and afterwards transferred onto nitrocellulose filters (Schleicher & Schüll/GE Healthcare, UK) with a pore size of 0.2 *μ*m with the wet blotting device “Mini-Protean II Cell and System” (BioRad, CA, US). Membranes were incubated with primary antibodies (Abs) diluted 1 : 1000–1 : 100 against HSF1, Hsp40, Hsp70A (C92F3A-5), Hsp70B′, Hsp90 (AC-88), Grp94 (9G10) all from Stressgen (distributed by Biomol, Hamburg, Germany), BclxL (2H12; Calbiochem/Merck-Millipore, Darmstadt, Germany), MelanA (A103; Sigma-Aldrich), or* beta*-actin (AC-74; Sigma-Aldrich), followed by HRP-conjugated horse anti-mouse IgG, goat anti-rabbit IgG, or goat anti-rat IgG Abs (all Cell Signaling Technology, UK) diluted 1 : 10000–1 : 2000. Detection was performed with the chemiluminescent Pierce ECL Western Blotting Substrate (ThermoFisher Scientific) on a high performance chemiluminescence film (GE Healthcare).

### 2.15. Luciferase Reporter Assay

Luciferase activity was measured using the luciferase assay system (Promega) according to the manufacturer's instructions, but utilizing 50 *μ*L of luciferase assay reagent and 10 *μ*L of cell lysate. Relative luminescence units (RLUs) were obtained with a Wallac Victor plate reader (PerkinElmer, MA, US). RLUs were normalized to the protein concentration as determined using the Pierce BCA*™* Protein Assay Kit (ThermoFisher Scientific).

### 2.16. Statistical Analyses

The significance of differences was determined by using either one-way ANOVA or two-way ANOVA and Bonferroni's Multiple Comparison* post hoc* test; *P* values < 0.05 were considered statistically significant.

## 3. Results

### 3.1. The “Modular Promoter” System

**Figure 1:**
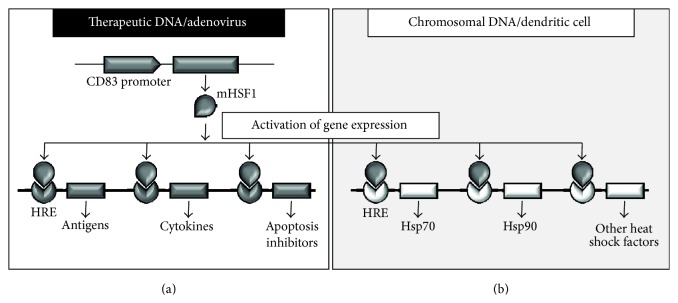
Schematic representation of the therapeutic DNA/adenovirus strategy and the effects of mHSF1 overexpression on endogenous heat shock response elements. (a) A constitutively active mutant heat shock factor 1 (mHSF1) will be expressed under the control of the human mDC-specific CD83 promoter. In turn mHSF1 binds to short heat shock response elements (HRE) of a second vector-encoded DNA driving expression of therapeutic transgenes (e.g., antigens, cytokines, or apoptosis inhibitors). (b) By overexpressing mHSF1 in the target cell, also endogenous HRE will become activated resulting in the expression of different heat shock proteins (Hsp), such as Hsp70 or Hsp90.

We considered two aspects to be important for the development of a combined promoter system, the so-called modular promoter (MP) system, to transcriptionally target DCs. On the one hand, the system should be highly specific for the targeted DC and on the other hand, the therapeutic transgenes should be expressed very efficiently. As there is only limited space for foreign DNA in plasmid and especially viral vectors, we envisioned a dual vector system, in which one vector expresses a mutated, constitutively active heat shock factor (mHSF) 1 [26] under the control of the human mDC-specific CD83 promoter [18] (Figure 1(a)). The mHSF1, in turn, will then induce simultaneous expression of different therapeutic transgenes contained in the second vector, such as tumor antigens, proinflammatory cytokines, or apoptosis inhibitors, by binding to the short heat shock response elements (HRE; Figure 1(a)). We hypothesized that the expression of tumor antigens in combination with different therapeutic proteins will subsequently enhance the capacity of these modified DCs to induce improved and potent antitumoral immune responses.

Of note, mHSF1 may also induce the expression of different endogenous heat shock proteins, such as Hsp70 or Hsp90, by binding to the cellular HRE (Figure 1(b)). Therefore, it was indispensable to examine the resulting effects of an overexpression of mHSF1 on DCs, especially regarding (i) expression of phenotypic cell surface maturation markers, (ii) toxicity, and (iii) function.

### 3.2. Specific Induction of the Short pHsp70B′_−29/−242_ Promoter in HeLa Cells

**Figure 2:**
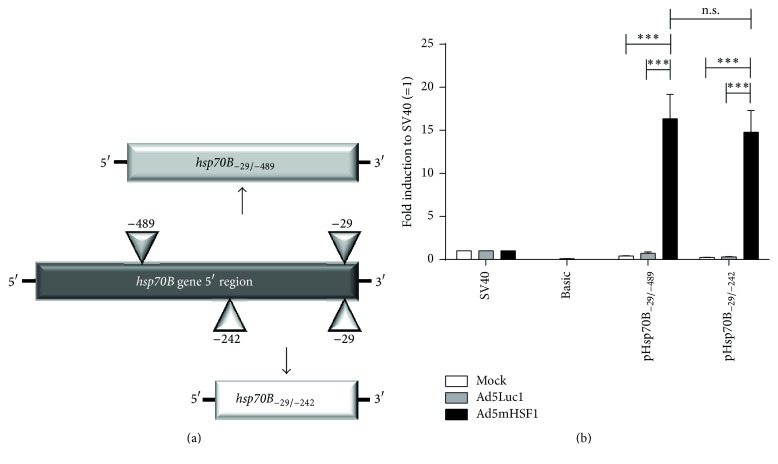
Generation and evaluation of two different Hsp70B heat shock response elements. (a) Plasmid vectors containing Hsp70B_−29/−489_ and Hsp70B_−29/−242_ were generated by digesting the human* hsp70B* gene 5′-region with HindIII/BamHI or HindIII/SmaI, respectively, followed by ligation into the pGL3Basic vector. (b) HeLa cells were transfected with 0.5 *μ*g of plasmid DNA of pGL3-SV40-promoter vector (“SV40”), promoterless pGL3-Basic vector (“Basic”), pHsp70B_−29/−489_, or pHsp70B_−29/−242_. The next day, cells were either left untreated (“Mock”) or transduced with adenoviruses Ad5Luc1 or Ad5mHSF1 at 300 TCID_50_/cell. Twenty-four hours later, cells were harvested and analyzed by luciferase reporter assays. Results are shown as fold induction relative to the individual pGL3-SV40-promoter control containing no virus, Ad5Luc1 or Ad5mHSF1. Data are mean ± SEM of three independent experiments. ^*∗∗∗*^ *P* < 0.001, n.s.: not significant (*P* > 0.05); bars without annotation are not significant (*P* > 0.05); two-way ANOVA with Bonferroni's Multiple Comparison* post hoc* test.

To develop a new and efficient dual promoter-based transcriptional DC-targeting strategy, we initially compared the activity of two different promoter fragments derived from the 5′ region of the human* hsp70B* gene. In 1985, Richard Voellmy isolated and characterized a 450 bp BamHI/HindIII fragment of a 70,000-dalton heat shock protein segment (Hsp70) involved in the control of transcription and translation of heat shock proteins [34]. Later, Schiller and colleagues reported this copy of the* hsp70* gene as the* hsp70B* gene [35]. Four cis-acting heat shock regulatory sequences (HSE 1–4) were described, which were supposed to contribute to the Hsp70B promoter activity. Additionally, HSE 1–3 (within a short fragment of ~200 bp in the 5′ nontranscribed region) were shown to be sufficient for optimal activity in human HeLa cells. To determine if the shorter element is sufficient, we cloned a −29/−489 bp and a −29/−242 bp fragment of the human* hsp70B* gene 5′ region (according to GenBank accession no. X13229) into the pGL3-Basic luciferase reporter vector resulting in pHsp70B_−29/−489_ and pHsp70B_−29/−242_, respectively (Figure 2(a)). Afterwards, plasmids were used to transfect HeLa cells. As a control we used the promoterless pGL3-Basic (“Basic”) and the pGL3-SV40-Promoter (“SV40”) vector. The next day, transcriptional activity was induced by the transduction of HeLa cells with an adenovirus encoding for a mutant constitutively active HSF1 (Ad5mHSF1). Transduction with Ad5Luc1 or Mock treatment served as controls. Twenty-four hours later, a luciferase reporter assay was performed and results were normalized relative to the respective pGL3-SV40-Promoter control. As shown in Figure 2(b), the Hsp70B_−29/−489_ and Hsp70B_−29/−242_ promoter were exclusively induced by Ad5mHSF1 up to 16-fold relative to the SV40 promoter, whereas there was no induction in the Mock sample or by the control virus. Furthermore, neither Ad5Luc1 nor Ad5mHSF1 influenced the transcriptional activity of the SV40 promoter and the Basic vector. Interestingly, there was no difference in the promoter activity when directly comparing the long Hsp70B_−29/−489_ with the short Hsp70B_−29/−242_ promoter, but looking closely at the Mock-treated versus the Ad5mHSF1-transduced cells for each promoter, we found an increase of up to 40 times for the longer promoter fragment and up to 64 times for the shorter one. Finally, the Hsp70B_−29/−242_ promoter was listed as the Hsp70B′ (HSPA6) promoter in the GenBank (accession no. NM_002155) in 2004.

Thus, we identified the Hsp70B′_−29/−242_ promoter to be highly and specifically induced in HeLa cells and for this reason it was used in the following experiments to generate the “modular promoter” system.

### 3.3. Multiple Therapeutic Transgenes Can Be Simultaneously Expressed under the Control of the Hsp70B′_−29/−242_ Promoter

**Figure 3:**
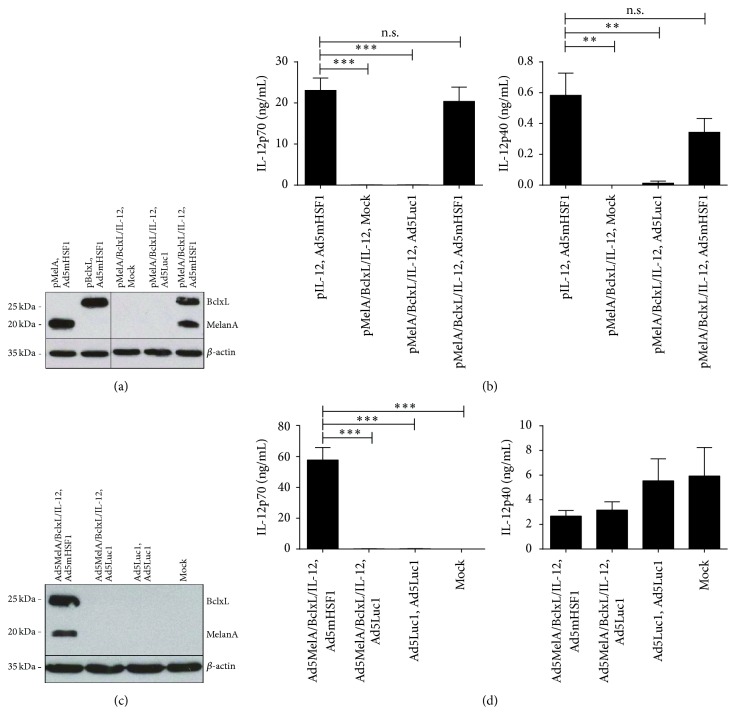
The constitutively active mHSF1 induces specific transgene expression in HeLa cells and human DCs. (a, b) HeLa cells were transfected with 0.7 *μ*g DNA of plasmid vectors expressing MelanA/MART-1 (“MelA”), BclxL or/and IL-12 under the control of the Hsp70B′_−29/−242_ heat shock response element as indicated. The next day, cells were either left untreated (“Mock”) or transduced with adenoviruses Ad5Luc1 or Ad5mHSF1 at 300 TCID_50_/cell. (c, d) Human immature DCs were cotransduced in the presence of the maturation cytokine cocktail with adenoviruses as indicated at a total amount of 500 TCID_50_/cell. (a–d) Twenty-four hours later, cell lysates were analyzed for BclxL and MelanA expression by Western Blot (a, c) and cell culture supernatants for content of IL-12p40 and IL-12p70 by ELISA (b, d). (a and c) Showing one representative experiment out of three; (b and d) data are mean ± SEM of three independent experiments with different donors. ^*∗∗*^ *P* < 0.01, ^*∗∗∗*^ *P* < 0.001, and n.s.: not significant (*P* > 0.05); bars without annotation are not significant (*P* > 0.05); one-way ANOVA with Bonferroni's Multiple Comparison* post hoc* test.

In order to prove our concept that mHSF1 can induce not only the expression of single transgenes but also the simultaneous expression of several transgenes under the control of the Hsp70B′_−29/−242_ promoter, we generated plasmid vectors containing either a Hsp70B′_−29/−242_-MelA/MART-1 (“pMelA”), a Hsp70B′_−29/−242_-BclxL (“pBclxL”), a Hsp70B′_−29/−242_-IL-12 (“pIL-12”), or the triple Hsp70B′_−29/−242_-MelA-Hsp70B′_−29/−242_-BclxL-Hsp70B′_−29/−242_-IL-12 (“pMelA/BclxL/IL-12”) expression cassette. HeLa cells were transfected with the above-mentioned vectors followed by transduction with Ad5mHSF1, Ad5Luc1, or Mock the next day. Twenty-four hours later cells were harvested, lysed, and separated by SDS-PAGE followed by Western Blotting using specific antibodies for MelanA/MART-1, BclxL, and beta-actin expression. As shown in Figure 3(a), MelanA and BclxL were highly expressed only after specific HSP70B′ promoter activation by Ad5mHSF1. Of note, the MelanA and BclxL proteins were equally well expressed from the triple plasmid pMelA/BclxL/IL-12. For the determination of the IL-12 concentration in the cell culture supernatants of cells analyzed in (a), IL-12p70- and IL-12p40- specific ELISA were performed (Figure 3(b)). Again, IL-12 production after transfection with pIL-12 or pMelA/BclxL/IL-12 was induced by Ad5mHSF1. Transduced HeLa cells secreted high amounts of the immunologic active IL-12p70 (23.06 ng/mL and 20.39 ng/mL, resp.) and much lower amounts of the homodimer IL-12p40 (0.58 ng/mL and 0.34 ng/mL, resp.). Moreover, there was almost no difference in the quantity of either IL-12p70 or IL-12p40 expressed from pIL-12 or pMelA/BclxL/IL-12, respectively.

Regarding our aim to insert the MP system into an adenoviral vector, we next generated Ad5MelA/BclxL/IL-12 containing an Hsp70B′_−29/−242_-MelanA/Hsp70B′_−29/−242_-BclxL/Hsp70B′_−29/−242_-IL-12 expression cassette. Adenovirus vectors contain several promoters within their own genome, for example, the early transcripts E1A-E4, as well as multiple recognition sites within these promoters for transcription factors. They often represent cis-acting DNA sequences that increase transcription, independent of their orientation and distance relative to the RNA start site [36]. To exclude the possible interference of these viral enhancers and upstream regulatory elements with the Hsp70B′ promoter of our system, we cotransduced human iDCs with Ad5MelA/BclxL/IL-12, Ad5mHSF1 or control virus Ad5Luc1 in the presence of a cytokine maturation cocktail. As a control, cells were matured in absence of an adenoviral vector (“Mock”). Twenty-four hours later, lysates of DCs were generated to perform Western Blot analyses to assess MelanA, BclxL, and beta-actin expression (Figure 3(c)). In addition, cell culture supernatants were analyzed by ELISA for the content of IL-12p70 and IL-12p40 (Figure 3(d)). As depicted there, only the cotransduction of Ad5MelA/BclxL/IL-12 with Ad5mHSF1 induced a specific transgene expression of MelanA, BclxL, and high amounts of secreted IL-12p70 (57.84 ng/mL). Furthermore, DCs transduced with Ad5MelA/BclxL/IL-12 produced less of the homodimer IL-12p40 in comparison to Mock or only Ad5Luc1 treated DCs (2.67 and 3.16 ng/mL versus 5.93 and 5.54 ng/mL, resp.).

In summary, we showed that the expression of multiple therapeutic transgenes under the control of the Hsp70B′ promoter can specifically be induced in HeLa cells, as well as in primary human DCs. Moreover, we demonstrated that the Hsp70B′ promoter acts in a highly specific manner, not only in a plasmid-based vector system, but particularly also in an adenoviral context.

### 3.4. Overexpression of mHSF1 Induces Expression of Heat Shock Proteins and Does Not Impair DC Function

**Figure 4:**
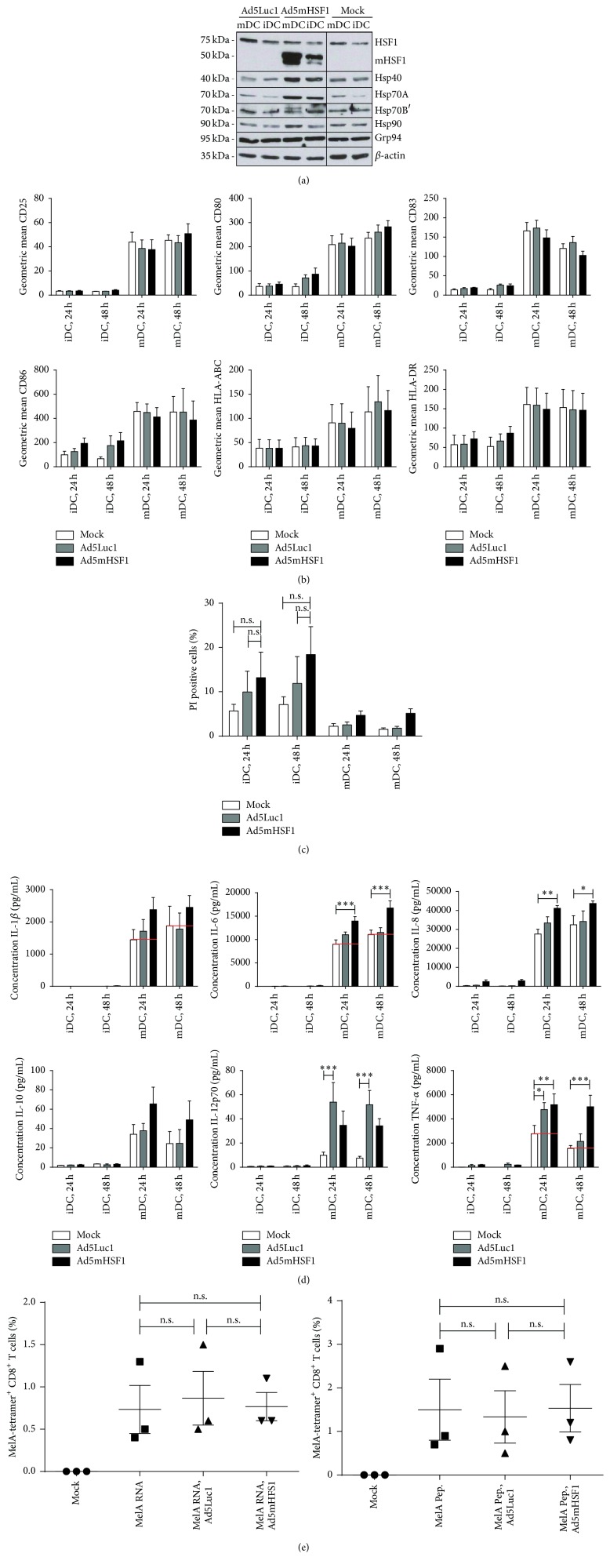
Overexpression of mHSF1 does not impair the phenotype and function of immature and mature DCs. Immature DCs were transduced with Ad5Luc1 or Ad5mHSF1 at 500 TCID_50_/cell or Mock-treated and were either left immature (“iDC”) or were matured (“mDC”) with a maturation cytokine cocktail (IL-1*β*, IL-6, TNF-*α*, and PGE_2_). (a) Western Blot analyses of cell lysates harvested 24 h after transduction on HSF1, mHSF1, Hsp40, Hsp70A, Hsp70B′, Hsp90, Grp94, and beta-actin protein expression. One representative experiment out of three is shown. (b, c) Flow cytometric analyses of iDCs and mDCs on CD25, CD80, CD83, CD86, HLA-ABC, and HLA-DR (b) as well as PI (c) 24 h and 48 h after transduction. Data are mean ± SEM of four independent experiments with DCs derived from different donors. (d) Cell culture supernatants derived from experiments shown in (b and c) were analyzed by cytometric bead array for the content of IL-1*β*, IL-6, IL-8, IL-10, IL-12p70, and TNF-*α*. The red line in (d) indicates background levels of respective cytokines derived from the cytokines present in the maturation cocktail. Data are mean ± SEM of three independent experiments. (e) Twenty-four hours after adenoviral transduction accompanied by maturation of DCs, cells were either transfected with wild-type MelanA RNA (left panel) or loaded with MelanA analogue peptide (right panel). Antigen-loaded DCs were cocultured with MACS-sorted autologous CD8^+^ T cells for seven days. Induction of antigen-specific CTLs was determined by HLA-A2-MelanA/iTag MHC class I-tetramer and anti-CD8-PC7 staining using a FC500 cytofluorometer. Data are mean ± SEM of three independent experiments with different donors. (b–e) ^*∗*^ *P* < 0.05, ^*∗∗*^ *P* < 0.01, ^*∗∗∗*^ *P* < 0.001, and n.s.: not significant (*P* > 0.05); bars without annotation are not significant (*P* > 0.05), one-way, (e) or two-way ANOVA (b–d) with Bonferroni's Multiple Comparison* post hoc* test.

As overexpression of mHSF1 is also expected to induce the activation of endogenous heat shock proteins thereby inducing a heat shock response of Ad5mHSF1 treated DCs, we addressed the influence of mHSF1 overexpression on DCs as follows. Immature DCs were transduced with Ad5mHSF1 or control virus Ad5Luc1 or Mock-treated either in the presence (mDC) or absence (iDC) of the maturation cocktail. Twenty-four and 48 hours later, analyses of DCs were conducted. First, regulation of heat shock proteins HSF1, Hsp40, Hsp70A, Hsp70B′, Hsp90, and Grp94 was determined in cell lysates of DCs by SDS-PAGE followed by Western Blotting (Figure 4(a)). Here, a clear increase in expression of heat shock proteins Hsp40 and Hsp70A was observed for iDCs and mDCs, whereas Hsp70B′ and Hsp90 was only induced in mDCs after Ad5mHSF1 treatment in comparison to Ad5Luc1 or Mock controls. HSF1 was only increased in Ad5Luc1 transduced mDCs, while mHSF1 was only found in DCs treated with Ad5mHSF1. Expression of Grp94 was unaffected by any treatment. Next, we examined the expression of typical cell surface activation markers including CD25, CD80, CD83, CD86, HLA-ABC (MHCI), and HLA-DR (MHCII) by FACS analyses (Figure 4(b)). As expected, all maturation markers of DCs treated with the cytokine cocktail (“mDC”) for 24 as well as 48 hours were upregulated in comparison to iDCs. Notably, treatment with any type of adenoviral vectors did not induce maturation of iDCs when compared to the Mock control. Furthermore, when DCs were matured during transduction (mDC), the adenoviral vectors did not influence the expression of the assessed surface markers. In addition to their activation status, DCs were also analyzed by FACS for their viability using propidium iodide (PI) staining. As shown in Figure 4(c), there was only a slight increase in the percentage of dead cells induced by the adenovirus treatment. The percentage of PI positive iDCs increased from 5.68% (Mock, 24 h) and 7.10% (Mock, 48 h) to 9.98% (Ad5Luc1, 24 h) and 11.90% (Ad5Luc1, 48 h) or 13.20% (AdmHSF1, 24 h) and 18.43% (AdmHSF1, 48 h), respectively. Matured DCs remained almost uninfluenced when compared with noninfected cells (2.20% and 1.55% PI^+^ cells after 24 h or 48 h, resp.). Transduction with Ad5Luc1 resulted in 2.55% (24 h) or 1.78% (48 h) and with Ad5mHSF1 in 4.68 (24 h) or 5.13% (48 h) PI positive DCs.

Next, we assayed the functionality of these DCs by evaluating their capacity to secrete proinflammatory cytokines including IL-1*β*, IL-6, IL-8, IL-12p70, and TNF-*α*, as well as the anti-inflammatory cytokine IL-10, by cytometric bead array 24 and 48 hours after treatment (Figure 4(d)). As the maturation cocktail contains IL-1*β*, IL-6, and TNF-*α*, Mock-treated DCs served as a control and yielded the background of the corresponding cytokine indicated by a red line in the graph. Interestingly, 24 and* 48* hours after infection, Ad5mHSF1-transduced mDCs showed a superior secretion of IL-1*β* (2381/*2451* pg/mL), IL-6 (13965/*16740* pg/mL), IL-8 (41154/*43674* pg/mL), IL-12p70 (35/*34* pg/mL), and TNF-*α* (5176/*5005* pg/mL) to Mock-treated mDCs (1443/*1877* pg/mL, 8993/*11063* pg/mL, 27581/*32420* pg/mL, 10/*7* pg/mL, and 2757/*1546* pg/mL, resp.). This was not the case for Ad5Luc1 transduced mDCs, where (with the exception of IL-12p70 and TNF-*α*) only a slight increase of cytokine production for IL-1*β* (1710/*1775* pg/mL), IL-6 (11038/*11519* pg/mL), and IL-8 (33398/*34201* pg/mL) could be observed either 24 or* 48* hours after transduction. Interleukin-10 is only secreted at very low amounts but slightly enhanced at both time points in supernatants of Ad5mHSF1 (65/*49* pg/mL) treated mDCs, but not of Ad5Luc1 (37/*24* pg/mL) treated ones, in comparison to the Mock control (34/*24* pg/mL). Immature DCs secreted almost no cytokines, independent of the treatment and time point at which they were analyzed. Finally, we assessed the functional ability of Ad5mHSF1 transduced mature DCs to prime autologous naïve CD8^+^ T cells. Therefore, iDCs were transduced with Ad5Luc1 or Ad5mHSF1 or were Mock-treated in the presence of the cytokine maturation cocktail the day before they were transfected with MelanA RNA or peptide-loaded with MelanA. Subsequently, DCs were cocultured with MACS-sorted CD8^+^ T cells for seven days and afterwards analyzed by staining with a HLA-A2-MelanA/iTag MHC class I-tetramer and an anti-CD8 antibody followed by flow cytometry. As shown in Figure 4(e), only antigen-loaded DCs induced the proliferation of MelanA-specific CD8^+^ T cells with analogue peptide-loaded DCs (1.5%) being twofold more potent than wild-type RNA-transfected (0.73%) DCs. Moreover, transduction with neither the control adenovirus (RNA: 0.87%, Pep: 1.34%) nor Ad-mediated overexpression of mHSF1 (RNA: 0.77%, Pep: 1.53%) impaired the capacity of antigen-loaded DCs to induce specific CTL responses, independent of the route of delivery.

From these data we conclude that neither transduction with the used adenoviral vectors* per se* nor the overexpression of mHSF1 interfered with the function of DCs. However, transduction with Ad5mHSF1 resulted in the upregulation of various heat shock proteins and proinflammatory cytokines by mDCs.

### 3.5. Specific Targeting of Mature DCs by a Combined CD83-Hsp70B′ Promoter System

**Figure 5:**
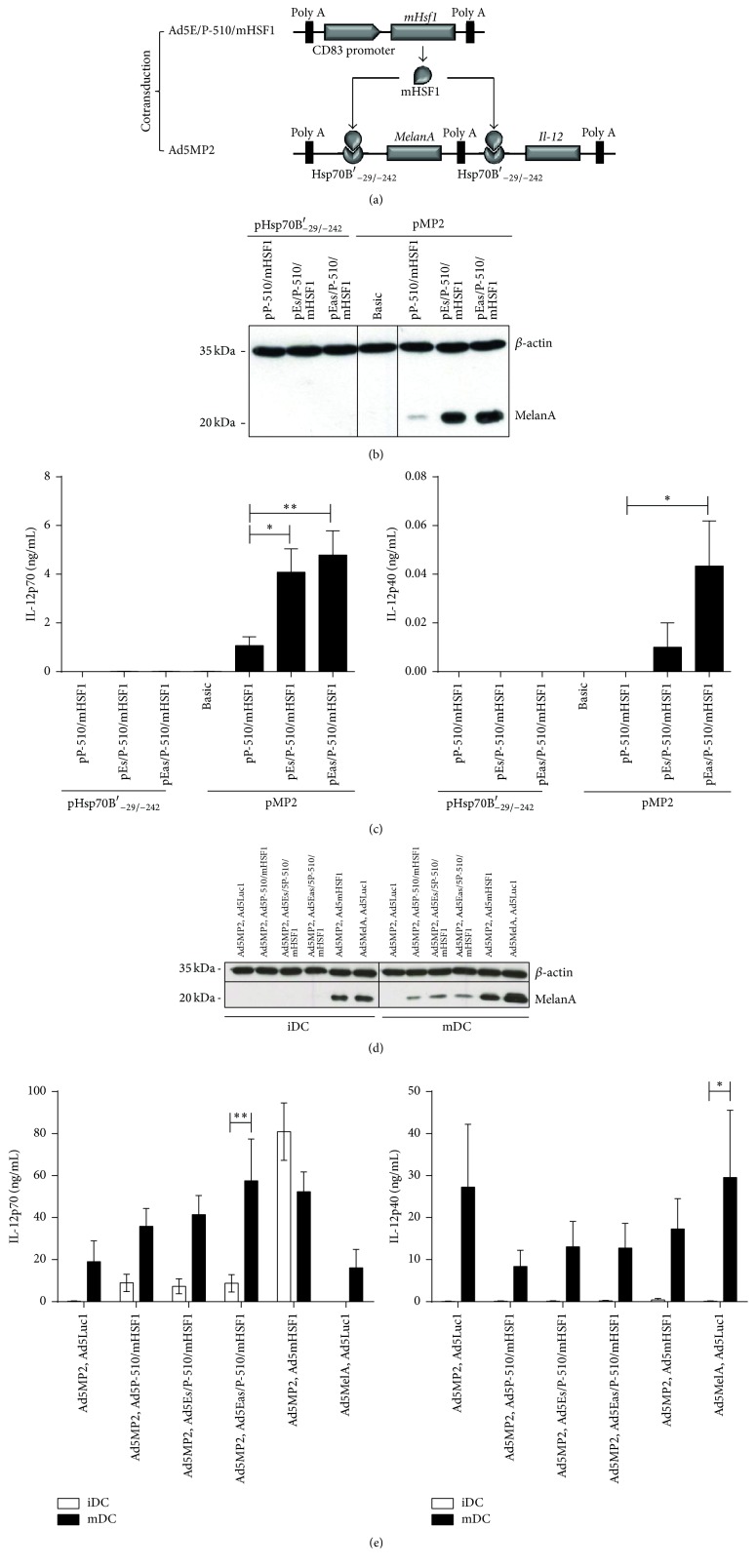
The “modular promoter (MP)” system enables highly efficient and specific expression of therapeutic transgenes in mature DCs. (a) Schematic outline of the dual vector “modular promoter” system. Ad5E/P-510/mHSF1 drives expression of mHSF1 under the control of the cell type- and maturation-specific human CD83 promoter, which in turn induces specific induction of therapeutic transgenes MelanA and Il-12 by binding to the Hsp70B′_−29/−242_ HRE encoded by Ad5MP2. (b, c) XS52 cells were cotransfected with a total amount of 2 *μ*g of plasmid vectors pP-510/mHSF1, pEs/P-510/mHSF1, or pEas/P-510/mHSF1 in combination with plasmid vectors encoding the HRE alone (“pHsp70B′_−29/−242_”) or the modular promoter system (“pMP2”). As a control, the empty pGL3-Basic vector (“Basic”) was used. (d, e) Human immature DCs were cotransduced with Ad5MP2 or Ad5MelA in combination with Ad5Luc1, Ad5mHSF1, Ad5P-510/mHSF1, Ad5Es/P-510/mHSF1, or Ad5Eas/P-510/mHSF1. Cells were either left immature or were matured by adding 0.1 ng/mL LPS. (b–e) Twenty hours after transfection, cell lysates were analyzed by Western Blot for expression of MelanA and beta-actin (b, d) and cell culture supernatants by ELISA for content of IL-12p40 and IL-12p70 (c, e). Either one representative (b, d) or data mean ± SEM from three independent experiments (c, e) is shown. ^*∗*^ *P* < 0.05; ^*∗∗*^ *P* < 0.01; bars without annotation are not significant (*P* > 0.05), one-way ANOVA (c) or two-way ANOVA (e) with Bonferroni's Multiple Comparison* post hoc* test.

To realize our final goal of transcriptionally targeting human mature DCs, we created new vectors where the expression of mHSF1 is driven by the human CD83 promoter, recently characterized by our group [18]. This tripartite promoter complex, consisting of a 510 bp upstream plus core promoter element (“P-510”) and a 185 bp enhancer (“E”) fragment, was shown to act in a cell type- and maturation-specific manner. To assess this, also in the context of the modular promoter system, we generated plasmid vectors (Figure 5(a)) containing mHSF1 and only the P-510 CD83 promoter (“pP-510/mHSF1”) or the P-510 plus the enhancer in either the sense or antisense direction (“pEs/P-510/mHSF1” or “pEas/P-510/mHSF1”). These were cotransfected into the murine DC-like cell line XS52 with plasmid vectors expressing MelanA/MART-1 and IL-12 under the control of the Hsp70B′_−29/−242_ HRE (“pMP2”; Figures 5(a)–5(c)). As a control pMP2 was replaced by (i) a vector comprising only the Hsp70B′_−29/−242_ promoter or (ii) the empty pLG3-Basic vector (“Basic”) (Figures 5(a) and 5(b)). Twenty-four hours later, cells were lysed for SDS-PAGE and Western Blotting for MelanA and beta-actin detection, while cell culture supernatants were analyzed by ELISA for the content of IL-12p70 and IL-12p40. As depicted in Figure 5(b), only cotransfection of pMP2 with pEs/P-510/mHSF1 or pEas/P-510/mHSF1 resulted in a strong and robust MelanA expression, while pP-510/mHSF1 without the enhancer showed only weak transgene expression. Accordingly, IL-12p70 secretion was not detectable in controls, at low levels in the context of pMP2 plus pP-510/mHSF1 transfected cells (1.07 ng/mL) and in large amounts by the combination of pMP2 with pEs/P-510/mHSF1 (4.08 ng/mL) or pEas/P-510/mHSF1 (4.79 ng/mL) (Figure 5(c)). Interleukin-12p40, however, was only detectable in supernatants derived from pMP2 plus pEs/P-510/mHSF1 (0.01 ng/mL) or pEas/P-510/mHSF1 (0.434 ng/mL) transfected XS52 cells.

Finally, we generated corresponding adenoviral vectors, that is, Ad5MP2, Ad5P-510/mHSF1, Ad5Es/P-510/mHSF1, and Ad5Eas/P-510/mHSF1. These were used together with Ad5Luc1 (negative control), Ad5MelA (positive control), or Ad5mHSF1 to cotransduce immature human DCs. DCs were then either left immature (“iDC”) or were matured with LPS for 20 hours (“mDC”) because this maturation stimulus induced the highest expression of the CD83 promoter ([18] and unpublished results). Afterwards, cell lysates were analyzed for expression of MelanA and beta-actin by Western Blot and supernatants by IL-12p70 and IL-12p40 ELISA. Clearly, MelanA expression was specifically induced only in mDCs after cotransduction of Ad5MP2 with adenoviruses containing the CD83 promoter-mHSF1 cassette (Figure 5(d)). Positive controls Ad5MP2 plus Ad5mHSF1, as well as Ad5MelA plus Ad5Luc1, revealed expression of the transgene in iDCs and mDCs, while negative controls did not show a signal for either. Similar results were obtained for cytokine-matured DCs (data not shown). Regarding IL-12p70, LPS-matured DCs, in contrast to cocktail-matured DCs, produced higher amounts in general (Figure 5(e)). Interestingly, in comparison to controls, that is, Ad5MP2 plus Ad5Luc1 (19.05 ng/mL) or Ad5MelA plus Ad5Luc1 (16.1 ng/mL), the amount of IL-12p70 expressed doubled when Ad5MP2 was cotransduced with Ad5P-510/mHSF1 (35.87 ng/mL), Ad5Es/P-510/mHSF1 (41.46 ng/mL), or Ad5Eas/P-510/mHSF1 (57.56 ng/mL). Cocktail-matured DC (data not shown) showed a lower, but specific, IL-12p70 production upon cotransduction of Ad5MP2 with Ad5P-510/mHSF1 (9.83 ng/mL), Ad5Es/P-510/mHSF1 (16.78 ng/mL), or Ad5Eas/P-510/mHSF1 (16.83 ng/mL). In comparison to LPS-matured DCs, immature DCs showed a lower expression of IL-12p70 (max. 8.93 ng/mL), while in the positive control (Ad5MP2 plus Ad5mHSF1), higher amounts of IL-12p70 were detected for both LPS-matured (52.3 ng/mL) and immature DCs (80.96 ng/mL). In contrast, IL-12p40 was mainly secreted by control-transduced LPS-matured DCs (max. 29.54 ng/mL), whereas LPS-matured DCs transduced with the MP adenoviruses showed less (max. 13.05 ng/mL) and iDCs no IL-12p40 irrespective of the adenovirus used.

Taken together, we have here demonstrated our modular promoter system to specifically target human immunogenic mature DCs to efficiently induce the expression of different therapeutic transgenes.

## 4. Discussion

One big challenge facing cancer immunotherapy is the generation of cell-specific treatment strategies. For* in vivo* DC-targeting, the restriction of transgene expression to this specific cell type is the main interest. Adenoviruses display several advantages for this targeted delivery of therapeutic transgenes such as tumor antigens and immune stimulatory proteins. They permit an extended expression of full length antigens within the transduced DC. In contrast to other targeting strategies, where peptides, proteins, or mRNAs are used, the cancer-proteins are synthesized over a longer period and processed by the DCs' own antigen-presentation machinery. As a consequence, long-lasting antigen-presentation to T cells is ensured without generating concerns regarding the breakdown of peptide/MHC complexes [37].

Melanoma is viewed as an “immunogenic” tumor type which is why here Ad-based and other strategies to improve DC-mediated immunotherapies have been used for the treatment of this disease. Moreover, malignant melanoma is the most common cause of mortality from skin cancer worldwide. It is refractory to irradiation and chemotherapy in late phases but rather amenable to immunological approaches [38]. Although great efforts have been made in recent years, antitumor DC-based vaccines rarely exceeded 15% of the objective clinical responses [39]. Hence, there is still a high medical need to develop new strategies to improve DC vaccination.

In this study, we present* in vitro* experiments using a combined CD83-Hsp70B′ promoter system (“modular promoter [MP]” system; Figures 1 and 5(a)) to specifically target mature DCs for the simultaneous expression of different therapeutic transgenes in the context of malignant melanoma. Therefore, we expressed a mutated HSF1 (mHSF1) with a deletion between amino acid positions 202–316 of wild-type HSF1, which has been shown to be constitutively active even in the absence of stress [26], under the control of the DC- and maturation-specific human CD83 promoter [18]. In turn, mHSF1 is used to drive simultaneous Hsp70B′-dependent therapeutic transgene expression. Heat shock promoters, particularly Hsp70 promoters, have often been used for gene therapy in the past and the Hsp70B′ promoter displays several advantages. First, it is induced only after severe stress, resulting in a lower basal expression compared to other* hsp70* genes [40, 41]. This is in accordance with our data, as these demonstrated the Hsp70B′ promoter to be induced only by mHSF1 and not to be sensitive to stress factors induced by lipofection (Figure 2(b)) or adenoviral transduction (Figure 3). Second, the moderate expression of Hsp70B′ was reported to be restricted to white blood cells like DCs, monocytes, and NK cells especially but to be nearly absent in other blood cells and tissues [42]. Third, the core Hsp70B′ promoter element is very short (213 bp) and showed a promoter activity 16 times higher than the strong SV40 promoter (Figure 2(b)). In addition, (m)HSF1 is not immunogenic as it is an ubiquitously expressed protein.

Interestingly, overexpression of mHSF1 did not influence the maturation status, the survival of immature and mature DCs, or their capability to prime naïve CD8^+^ T cells in an antigen-specific manner whereas heat treatment of these DCs did result in the upregulation of maturation markers and increased capability to prime naïve CD8^+^ T cells [27]. However, mHSF1 overexpression or adenoviral transduction of mDCs per se induced elevated levels of proinflammatory cytokines including IL-1*β*, IL-6, IL-8, IL-12p70, and TNF-*α* in cell culture supernatants. We also found Hsp40 and Hsp70A to be upregulated in cell lysates of iDCs and mDCs, whereas Hsp70B′ and Hsp90 were only induced in mDCs. Heat shock proteins such as Hsp40 and Hsp70 have been described to act as chaperones and be involved in the proper folding of proteins as well as in the prevention of the formation of nascent protein aggregations thereby keeping the proteins in a substrate-grabbable state. Others, like Hsp90, have been demonstrated to contribute to antigen-presentation on MHC I [43–45]. In this regard, heat shock proteins also became the focus of attention in the vaccine research area. Exogenous Hsp70 and Hsp90 have been reported to induce secretion of cytokines like GM-CSF, IL-1*β*, IL-6, IL-12, and TNF-*α* and to upregulate cell surface maturation markers CD83, CD86, or CD40 as well as to enhance the migratory capacity of DCs [46–48]. Induction of heat shock proteins, on the other hand, is ensued by the induction of HSF1 [49]. Besides its role as a transcription factor in stressed cells, HSF1 is involved in a multitude of physiological processes such as cell metabolism, gametogenesis, aging, insulin signaling, and cancer progression [24, 25]. Regarding the latter, HSF1 was shown to act on cancer development by regulating tumor cell proliferation, antiapoptosis, epithelial-mesenchymal transition (EMT), migration, invasion, and metastasis [50]. Interestingly, almost nothing is known about the influence of HSF1 or mHSF1 overexpression on human DCs. Thus, at least to our knowledge, this is the first study indicating that overexpression of mHSF1 does not impair DC maturation and function, although varying reports on the DC's responsiveness to heat shock were published [27, 51–53]. Solely Ostberg and colleagues reviewed that effects such as the induction of maturation or stimulation of T cells, induced by mild thermal stress, are not dependent on HSF1-mediated transcriptional events in murine bone marrow derived DCs [54]. The mHSF1 used in the present study however was also overexpressed by Xia and colleagues in HeLa cells [55]. As a consequence, HeLa cells, which are normally relatively insensitive to Fas-mediated killing, became sensitive to Fas-induced apoptosis, thereby providing a new target for Fas-based antitumor vaccines.

Encouraged by these results, we generated the modular promoter system to transcriptionally target mature DCs. To perform first proof of principle, we successfully expressed several therapeutic transgenes under the control of the core Hsp70B′ promoter element, which we could specifically induce with CMV promoter-driven mHSF1 both in HeLa cells by plasmid transfection and in primary mature human DCs by adenoviral vectors (Figure 3). In a second step, we developed a two-vector-based system, where we set mHSF1 under the control of the human DC- and maturation-specific CD83 promoter to express MelanA and IL-12p70 (Figure 5(a)). The advantage of MelanA is its widespread expression on melanocytic cells and its ubiquitin-mediated proteasome-dependent degradation leading to the presentation of the antigen via MHC class I [56]. Interleukin-12p70, on the other hand, enhances the cytotoxic activity of CD8^+^ CTLs and NK cells [57]. Moreover, exogenous expression of IL-12p70 is able to overcome the inhibitory effect of PGE_2_ on IL-12p70 production of DCs matured with the standard cytokine maturation cocktail used not only* in vitro* but also* in vivo* in clinical trials [58]. Of note, the combined CD83-Hsp70B′ promoter system not only facilitated specific transcriptional targeting of mature immunogenic DCs but also guaranteed high expression of the respective transgenes (Figure 5) without affecting endogenous CD83 expression in maturing DCs (data not shown).

To date, only very few promoter systems have been proven suitable for the transcriptional targeting of DCs. The group of Ross et al., for instance, demonstrated the promoter of the cytoskeletal fascin protein to induce specific transgene expression in mature murine DCs after gene gun-mediated DNA immunization [59]. Similar results were also reported by Sudowe et al. to induce type 1 immune responses [59, 60]. In the context of a lentiviral vector system, Dresch and colleagues described the 5′ untranslated region from the* DC-STAMP *gene as a suitable promoter region to yield long-term and cell-selective transgene expression in murine DCs* in vivo* [61]. Several cell type-specific promoters have been reported in the past to target murine DCs* in vitro* and* in vivo;* however here we report the first promoter system allowing the specific targeting of mature human DCs. Regarding its future prospects for cancer therapy, the CD83-Hsp70B′ promoter system offers several interesting features. Due to its modular composition, the system is highly flexible and hence not restricted to a specific tumor entity or therapeutic application. On the one hand, the therapeutic transgenes encoded by one adenoviral vector could be replaced by any gene of interest, even mutated cancer- and patient-specific TAAs. On the other hand, it would be possible to use an even more specific promoter or one with different cell-specificity, for the expression of the mHSF1 within the other vector. This modular composition would in theory allow the* in vivo *targeting of any combination of transgenes to any cell type although this requires substantial additional preparatory experiments. Moreover, by combining transductional targeting of adenoviruses to dendritic cells via bispecific diabodies or single-chain antibodies incorporated into the virus capsid, a further increase of targeting specificity might be achieved.

Finally, since CD83 is not expressed by immature but is highly induced in mature DCs, the applied CD83 promoter complex represents an ideal candidate for transcriptional targeting of mature DCs* in vivo* and could set the stage for next generation* in situ* vaccination strategies. This should be particularly effective and safe, as it assures selective antigen expression in mDCs while avoiding expression in tolerogenic iDCs for the first time. These promising perspectives, however, need to first be analyzed and explored in further preclinical investigations.

## 5. Conclusions

In this study, we present the initial* in vitro* experiments showing that a combined two-vector CD83-Hsp70B′ promoter system is able to transcriptionally target human mature DCs for the first time with a highly specific and effective expression of a panel of potential therapeutic transgenes. Moreover, we first demonstrate that overexpression of mHSF1 does not interfere with DC maturation, viability, and function. Hence, this study provides valuable new insights for the development of a safe, modular, highly flexible, and hence patient-tailored vaccination protocol for* in vivo* targeting of DCs, but also other efficient genetic therapies in the future.
